# Analysis of the Mass Transfer Kinetics of Dealuminated Jellyfish During Ethanol Pickling Process

**DOI:** 10.3390/foods14173067

**Published:** 2025-08-30

**Authors:** Yihe Zhang, Pengfei Yi, Jingkang Xu, Kui You, Xinghua Li, Jiajun Ren, Heyang Bai, Caihua Ma

**Affiliations:** 1 School of Chemical, Oceanic and Life Sciences, Dalian University of Technology, Panjin 124000, China; zhangyihe@mail.dlut.edu.cn (Y.Z.); 913482740@mail.dlut.edu.cn (P.Y.); jingkangxv@mail.dlut.edu.cn (J.X.); lierde@mail.dlut.edu.cn (X.L.); jiajunr@mail.dlut.edu.cn (J.R.); bhy790447702@mail.dlut.edu.cn (H.B.); 2 School of Economics, Ocean University of China, Qingdao 266100, China; hd20072011@163.com

**Keywords:** jellyfish alum removal, ethanol pickling, mass transfer kinetics

## Abstract

The main quality and safety issue in processing salted jellyfish for food is excessive aluminum. After dealumination, problems such as a low quality and short shelf life may occur. A method for reprocessing dealuminated jellyfish that can maintain quality and yield bactericidal effects is necessary. Alcohol provides astringent protein and bactericidal effects, and ethanol is safe and nontoxic. It can be added as needed in food production. The optimal processing conditions were determined by studying the mass transfer and quality changes in dealuminated jellyfish at different ethanol concentrations. The results revealed that both the ethanol concentration and pickling time significantly affected the mass transfer changes of substances in the pickling process for dealuminated jellyfish. The total mass of dealuminated jellyfish decreased with increasing ethanol concentration, whereas the ethanol content increased. The changes were more obvious at the early stages of pickling, and then tended to flatten out. The diffusion coefficient was the highest for the 45% pickling solution, and the texture characteristics were similar to those of edible jellyfish, thus rendering this solution more suitable for dealuminated jellyfish ethanol soaking. In addition, the mass transfer model for various substances in the pickling process for dealuminated jellyfish exhibited a suitable linear correlation with time, which can be effectively applied.

## 1. Introduction

Jellyfish is a favorite seafood of the Chinese people, it is widely distributed in the four major sea areas of China and is highly valued for their economic and nutritional benefits [1]. Currently, jellyfish products in the market can be categorized into two main types: salt-preserved and ready-to-eat products. Owing to their inherent characteristics and the temperature during the capture season, fresh jellyfish is prone to autolysis if not promptly processed after harvesting. In the industrial sector of China, the ‘three-alum-two-salt’ method is employed to process fresh jellyfish, which involves removing a significant amount of water to produce salt-preserved jellyfish. This method ensures a long shelf life and easy storage, may maintain the texture of jellyfish well, and yielding a traditional jellyfish product [2]. However, the traditional ‘three-alum-two-salt’ processing method results in salt-preserved jellyfish with high aluminum contents. Studies have revealed that aluminum is a harmful metal that can accumulate in the human body, causing chronic toxic effects [3]. Ready-to-eat jellyfish products in the market are typically processed by removing salt and alum from jellyfish, and adding food preservatives, cutting, seasoning, and packaging. These products often suffer from the widespread use of preservatives, inadequate sterilization processes, and difficulty in quality control [4]. Clearly, existing technologies have limitations, and developing methods for better stabilizing jellyfish and avoiding pathogen contamination after dealumination could represent a huge objective with public health benefits.

Alcohol, a broad-spectrum bacteriostatic agent, is widely applied in food preservation. Alcohol molecules can be directly incorporated into the food matrix through diffusion, or they can migrate into the food by penetrating into edible carrier materials [5]. The transfer of marinade and water in muscle samples in the pickling process is complex, and depends on various mass transfer mechanisms, with diffusion as the most significant process [6]. This is due to the concentration and osmotic pressure differences between the muscle and marinade, as well as the concentration gradient within the muscle [7]. This process is typically described by Fick’s second law, and the most well-known phenomenological model for diffusion is the Crank model [8], which consists of solutions to Fick’s laws of diffusion for different geometries, boundary conditions, and initial conditions. This model has been adopted by many authors (Andrés et al. [9]; Barat et al. [10]; Boudhrioua et al. [11]; Fuentes et al. [12]; Fuentes et al. [13]; Gallart-Jornet et al. [14]; Gallart-Jornet et al. [15]; Wang et al. [16]).

Most studies on the mass transfer kinetics during pickling have focused on salted meat products, whereas research on ethanol pickling has been almost nonexistent. This study aimed to explore the infiltration laws and macroscopic expression models during the pickling process of using edible alcohol to pickle dealuminated jellyfish alcohol production. In addition, this study aimed to bridge the gap in theoretical research and guide process control in practical production.

## 2. Materials and Methods

### 2.1. Experimental Materials

Saline jellyfish was purchased from the seafood market in Yingkou city, Liaoning Province. Sulfuric acid, nitric acid, anhydrous ethanol, citric acid, potassium dichromate, and zeolite were all of analytical purity.

### 2.2. Instruments and Equipment

The following instruments and equipment were used in this study: a 101-1AB electric heating drying oven, Tianjin Taisite Instrument Co., Ltd., Tianjin, China, an EST200-4 electronic balance, Shenyang Tianping Instrument Co., Ltd.; Shenyang, China, a UV-5800 UV–visible spectrophotometer, Shanghai Yuanxi Instrument Co., Ltd., Shanghai, China; a DK-S26 electric heating constant-temperature water bath, Shanghai Jinghong Experimental Equipment Co., Ltd., Shanghai, China, a pipette gun, Dalongxingchuang Experimental Instruments Co., Ltd., Beijing, China; a TMS-Pro texture analyzer, FTC Corporation, Washington, DC, USA; and a 7900X inductively coupled plasma mass spectrometer, Shanghai Agilent Technologies, Inc., Shanghai, China.

### 2.3. Experimental Methods

#### 2.3.1. Sample Processing and Sampling

The cleaned salted jellyfish skin was cut into several cylindrical jellyfish pieces with a diameter of 5.5 cm, each weighing (35 ± 2) g, by using a mold. The jellyfish was soaked in a 0.1% citric acid solution at a ratio of 1:5 for 1 h to remove aluminum. After soaking, the surface of the jellyfish was rinsed with clean water to remove any aluminum residue and acid, after which it was rinsed with distilled water. The surface moisture of the sample was wiped off. The jellyfish pieces were weighed, after which they were added to 35 g of a 35%, 45%, 55%, 65%, or 75% ethanol solution. The mixture was sealed and allowed to stand at 20 °C. Samples were collected at 0, 1, 3, 5, 7, 9, 18, and 24 h to measure the selected indicators.

#### 2.3.2. Water Content Determination

The water content was determined with reference to GB5009.3-2016 (National Food Safety Standard for Determination of Water in Food) [17], via the direct drying method.

#### 2.3.3. Alcohol Content Determination

The alcohol content was determined according to the potassium dichromate colorimetric method of Jianlou Mou [18] with minor modifications. After the sample surface was wiped dry with filter paper, 10 g of jellyfish was weighed and placed in a 500 mL distillation flask. Two hundred milliliters of water was added, the distillation apparatus was connected, and 50 mL of the solution was distilled. A 0.5 mL aliquot of the distilled solution was transferred to a 25 mL cuvette. Three milliliters of a 25% potassium dichromate solution and 5 mL of concentrated sulfuric acid were added, and ultrapure water was added to achieve a total volume of 15 mL. The mixture was shaken well, a test tube stopper was added, and the mixture was heated in a boiling water bath for 10 min. The mixture was then removed and cooled in running water. The absorbance was measured at 600 nm, and the ethanol content in the distilled solution (0.5 mL) was calculated via a standard curve. Then, the ethanol content in the sample was calculated.

#### 2.3.4. Total Mass Determination

The sample was removed, and surface water was removed with filter paper. The mass was measured with a balance.

#### 2.3.5. Determination of the Aluminum Content

The aluminum content was determined according to GB5009.268-2016 (National Food Safety Standard Determination of Multiple Elements in Food) [19], namely, via inductively coupled plasma emission spectrometry (the second method noted in the standard).

#### 2.3.6. Texture Measurement

The salted jellyfish skin was soaked in clean water for 6–8 h, and the water was changed multiple times throughout the process until no obvious salty taste remained. The end product is considered edible jellyfish. The sample to be tested was cut into round pieces with a diameter of 2.5 cm and soaked in water for 2 h at a material-to-liquid ratio of 1:20 to remove ethanol and achieve ready-to-eat conditions. After surface moisture was wiped off, the texture was determined. A disc-shaped probe was adopted to conduct texture profile analysis (TPA)-mode sample testing. The test conditions were as follows: the pretest rate was 60 mm/min, the test rate was 60 mm/min, the posttest rate was 60 mm/min, the compression deformation ratio was 50%, the interval time was 5 s, the lifting height was 20 mm, and the triggering force was 5.0 N.

#### 2.3.7. Calculation of the Total Mass, Water Content and Ethanol Content Changes in the Jellyfish

The total mass change $\Delta M_{t}^{\left(o\right)}$, water content change $\Delta M_{t}^{\left(w\right)}$, and ethanol content change $\Delta M_{t}^{C_{2}H_{6}O}$ in the jellyfish were calculated as follows ([6,14]):

(1) $$\Delta M_{t}^{\left(o\right)}=\;\frac{M_{t}^{o}-M_{0}^{o}}{M_{0}^{o}}\;\times \;100\%$$

(2) $$\Delta M_{t}^{\left(w\right)}=\frac{\;M_{t}^{o}X_{t}^{\left(w\right)}-M_{0}^{o}X_{0}^{\left(w\right)}}{M_{0}^{o}}\;\times \;100\%$$

(3) $$\Delta M_{t}^{C_{2}H_{6}O}=\frac{M_{t}^{o}X_{t}^{C_{2}H_{6}O}-M_{0}^{o}X_{0}^{C_{2}H_{6}O}}{M_{0}^{o}}\;\times \;100\%$$

where $M_{0}^{o}$ and $M_{t}^{o}$ denote the jellyfish mass values at times 0 and *t*, respectively; $X_{0}^{\left(w\right)}$ and $X_{t}^{\left(w\right)}$ denote the water contents in the jellyfish at times 0 and *t*, respectively; and $X_{0}^{C_{2}H_{6}O}$ and $X_{t}^{C_{2}H_{6}O}$ denote the ethanol contents in the jellyfish at times 0 and *t*, respectively.

#### 2.3.8. Calculation of the Ethanol Content in the Jellyfish Aqueous Phase

In the pickling process, the primary mass transfer flux involves the transport of water-soluble compounds, primarily ethanol, which occurs in the aqueous phase of the sample [10]. On the basis of Equation (4) [6], the ethanol content in the jellyfish aqueous phase $Z^{C_{2}H_{6}O}$ was estimated by measuring the moisture content $X^{\left(w\right)}$ and ethanol content $X^{C_{2}H_{6}O}$ in the jellyfish.

(4) $$Z^{C_{2}H_{6}O}=\frac{X^{C_{2}H_{6}O}}{X^{\left(w\right)}+X^{C_{2}H_{6}O}}\;\times \;100\%$$

#### 2.3.9. Describing the Changes in Jellyfish During Ethanol Pickling by an Empirical Equation

Equation (5) is an empirical equation that accounts for Fickian diffusion ([20,21]), and it can be applied to experimental data to obtain the kinetic constant of overall mass transfer [9].

(5) $${\Delta M}_{t}^{i}\;=\;1\;+\;k_{1}\;+\;k_{2}t^{0.5}$$

where ${\Delta M}_{t}^{i}$ denotes the change in the total mass $\Delta M_{t}^{\left(o\right)}$, water content $\Delta M_{t}^{\left(w\right)}$, or ethanol content $\Delta M_{t}^{C_{2}H_{6}O}$ in the jellyfish.

#### 2.3.10. Pickling Equilibrium Equation

When examining the equilibrium stage of the pickling process, the ethanol content in the muscle water phase ${Zₑ}^{C_{2}H_{6}O}$ is assumed to be equal to that in the pickling solution ${yₑ}^{C_{2}H_{6}O}$ [22]. This can be calculated via the mass ratio of the initially dealuminated jellyfish to the pickling solution $\frac{M_{0}^{\left(SD\right)}}{M_{0}^{\left(SS\right)}}$, as well as the water and ethanol contents ($X_{0}^{\left(w\right)}{,\;X}_{0}^{C_{2}H_{6}O},\;y_{0}^{\left(w\right)},\;and\;y_{0}^{C_{2}H_{6}O}$) in both the initially dealuminated jellyfish and the pickling solution, as expressed in Equation (6) [21].

(6) $${Zₑ}^{C_{2}H_{6}O}={yₑ}^{C_{2}H_{6}O}=\frac{\frac{M_{0}^{\left(SD\right)}}{M_{0}^{\left(SS\right)}}X_{0}^{C_{2}H_{6}O}+y_{0}^{C_{2}H_{6}O}\;}{\frac{M_{0}^{\left(SD\right)}}{M_{0}^{\left(SS\right)}}\;\left(X_{0}^{\left(w\right)}+X_{0}^{C_{2}H_{6}O}\right)+\left(y_{0}^{\left(w\right)}+y_{0}^{C_{2}H_{6}O}\right)}\times 100\%$$

#### 2.3.11. Effective Diffusion Coefficient 

The effective diffusion coefficient was determined by using the changes in ${Z_{t}}^{C_{2}H_{6}O}$ and ${y_{t}}^{C_{2}H_{6}O}$ values during the pickling process. Calculated based on the integral solution of condition a semi infinite plate and short pickling time in Fick equation (Equation (7)) for a semi-infinite plate and a short soaking time [8]. The equation includes an independent term K, which represents the influence of fluid dynamics or any other mass transfer phenomenon at the start of the process, thus allowing for adjustments in the deviation from the origin of the coordinate system.

(7) $$1-{Y_{t}}^{C_{2}H_{6}O}=1-\frac{{Z_{t}}^{C_{2}H_{6}O}-{y_{t}}^{C_{2}H_{6}O}}{{Z_{0}}^{C_{2}H_{6}O}-{Z_{e}}^{C_{2}H_{6}O}}=2{\times \left[\frac{D_{e}\times t}{\pi \times l^{2}}\right]}^{0.5}+K$$

where ${Y_{t}}^{C_{2}H_{6}O}$ denotes the mass transfer driving force between the jellyfish aqueous phase and the ethanol solution; ${Z_{0}}^{C_{2}H_{6}O}{{,\;Z}_{t}}^{C_{2}H_{6}O},\;{{\;and\;Z}_{e}}^{C_{2}H_{6}O}$ denote the ethanol contents in the jellyfish aqueous phase at time 0, at time t, and at equilibrium in the pickling process, respectively; ${y_{t}}^{C_{2}H_{6}O}$ denotes the ethanol content in the pickling solution aqueous phase at time t of the pickling process; $D_{e}$ denotes the effective diffusion coefficient, m^2^/s; and l denotes the thickness of half of the jellyfish block, m.

### 2.4. Statistical Analysis

All experiments were conducted in triplicate, and the results are expressed as mean values ± standard deviations. The experimental data were processed using Excel 2019, and graphs were generated in Origin 2024. One-way analysis of variance (ANOVA) was performed by using IBM SPSS Statistics 26.0, and the difference was considered significant (*p* < 0.05).

## 3. Results and Discussion

### 3.1. Changes in the Total Mass, Ethanol Content, and Water Content in the Jellyfish After Ethanol Pickling

**Figure 1:**
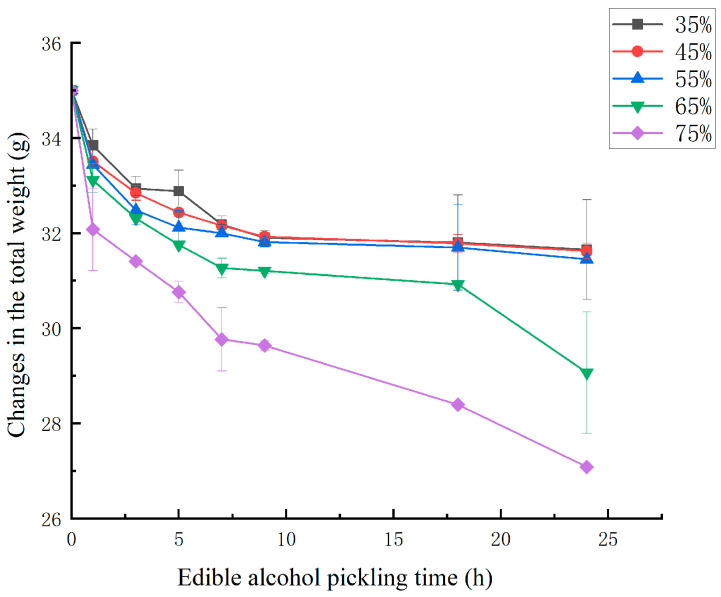
Changes in the total weight in dealuminated jellyfish during pickling for different ethanol concentrations.

In the pickling process, the complex cellular structure of food serves as a semipermeable membrane, since the membrane responsible for osmotic transport is not entirely selective, other solutes in the cells can also penetrate into the osmotic solution. In this process, the ethanol and water contents and weight change, eventually reach equilibrium ([23,24]). Figure 1 shows the weight changes of the dealuminated jellyfish in the ethanol-soaking process. The total weight changes in the pickling process were significantly influenced by the ethanol concentration (*p* < 0.05) and pickling time (*p* < 0.05). The figure shows that at the early stages of pickling, the weight of the jellyfish blocks first decreased rapidly, and then gradually slow down. The initial rapid weight change may be due to weight loss caused by the dissolution of salts and soluble substances in the dealuminated jellyfish blocks, combined with the increase in water due to fluid dynamics mechanisms [21]. After 9 h of pickling at concentrations of 35%, 45%, and 55%, the weight values of the jellyfish blocks remained similar and almost unchanged, whereas at concentrations of 65% and 75%, there were still decreases in mass, which may have been related to the increased protein denaturation and muscle damage that were caused by exposure to ethanol at high concentrations. Overall, the higher the concentration of the pickling solution is, the greater the weight reduction, indicating that low-concentration pickling helps increase the yield of jellyfish.

**Figure 2:**
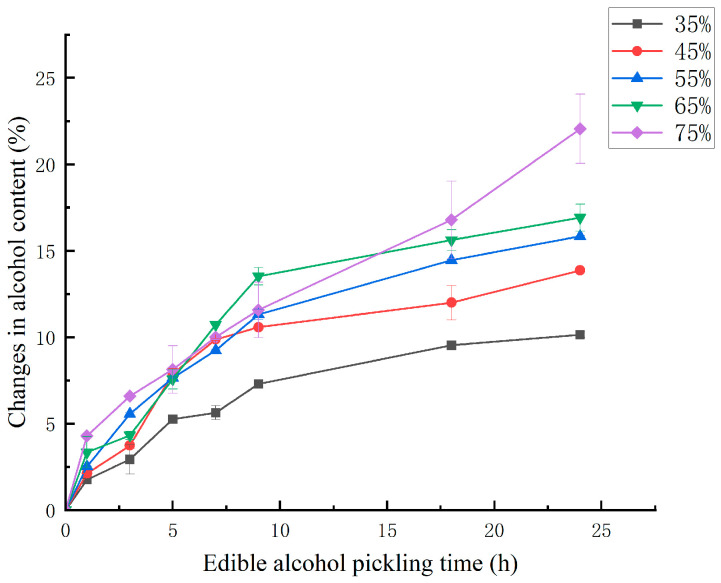
Changes in the ethanol content in dealuminated jellyfish during pickling for different ethanol concentrations.

Figure 2 shows the changes in the ethanol content in dealuminated jellyfish during ethanol pickling. The ethanol concentration and pickling time significantly affected (*p* < 0.05) the amount of ethanol penetration content in dealuminated jellyfish. As shown in the figure, the ethanol content in the jellyfish pieces at all concentrations increased with increasing pickling time, and the rate of change in the ethanol content was relatively high at the initial stage of pickling, which occurred mainly because the mass transfer driving force for diffusion mainly comes from the concentration gradient between the material and the solution. At the initial stage of pickling, the ethanol content in the pickling solution was much greater than that in the jellyfish tissues, therefore a significant difference in osmotic pressure occurred, and this difference in osmotic pressure prompted the diffusion of ethanol from the pickling solution into the jellyfish tissues, which resulted in a rapid increase in the ethanol content [25]. At the later stages of pickling, the internal and external osmotic pressures gradually decreased due to continuous diffusion, the mass transfer drive decreased, the diffusion rate decreased as a result, and the rate of change in the ethanol content decreased. When the internal and external osmotic pressures of the jellyfish tissues were equal, ethanol diffusion and water infiltration reached a dynamic equilibrium, and the change in the ethanol content stabilized. As expected, the solute content in the pickling agent increases, and the solute content in the sample also increases. These findings are consistent with those reported by Gou, Comaposada [26], Arnau [27], and Telis et al. [28].

**Figure 3:**
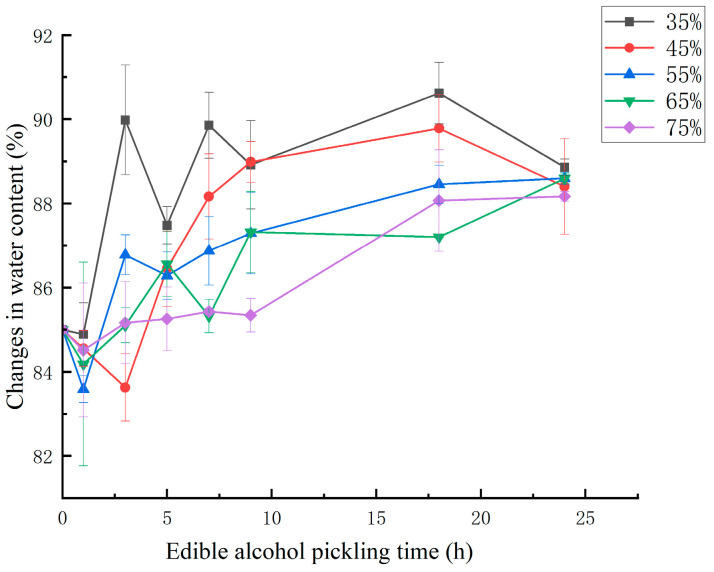
Changes in the water content in dealuminated jellyfish during pickling for different ethanol concentrations.

In the pickling process, the characteristics of muscle tissue changed due to variations in the concentration of the pickling solution. The pickling solution induced changes in muscle proteins, which led to changes in texture and water retention [29]. Figure 3 shows the changes in water content in the pickling process of jellyfish. The figure shows that the water content in the jellyfish blocks did not significantly change over time or with changes in the ethanol concentration. The water content initially decreased, but then increased, and it fluctuated between 85% and 90%. The initial outward diffusion of water was due to the higher osmotic pressure of the pickling solution, due to the inherent salt content of dealuminated jellyfish, the salt in the tissues will dissolve into the solution during the ethanol pickling process, resulting in a decrease in salt content. Giaver [30] reported that muscle tissue undergoes fibrousexpansion at lower salt concentrations (<50 g/L), the tissue water content increases when the sodium chloride concentration varies between 5 and 200 g/L, andwater loss occurs above 330 g/L. It is hypothesized that the salt content in the jellyfish in the pickling process remains less than 200 g/L, which explains the observed increase in water content. This observation agrees with earlier reports, which documented increased water retention in beef (Medyski, Pospiech, & Kniat, [31]), rabbit muscle fibers (Offer & Trinick, [32]), and pork tissue (Graiver et al. [33]). The increase in the water-holding capacity is attributed to the lateral expansion of myofibrils, which is associated with protein solubilization. The increased water binding and hydration of muscle fibers are typically attributed to the increased electrostatic repulsion between myofibril filaments, and the unfolding and swelling of the protein structural matrix, leading to the lattice expansion of the filaments to retain water.

### 3.2. Calculation of the Effective Diffusion Coefficient

The theoretical alcohol content at the equilibrium of pickling was calculated according to Equation (6), compare it with the actual measured alcohol content after 48 h of pickling, determine whether the pickling endpoint has been reached, and thus determining the pickling time [33]. As indicated in Table 1, the equilibrium values for both the 35% and 45% ethanol pickling solutions are very close, which indicates that after 48 h of pickling, the two soaking solutions had largely reached the equilibrium point. In the other ethanol concentration groups, the theoretical equilibrium value is slightly greater than the actual measured value, which suggests that after 48 h of pickling, the pickling process was still ongoing but was very close to the endpoint.

**Figure 4:**
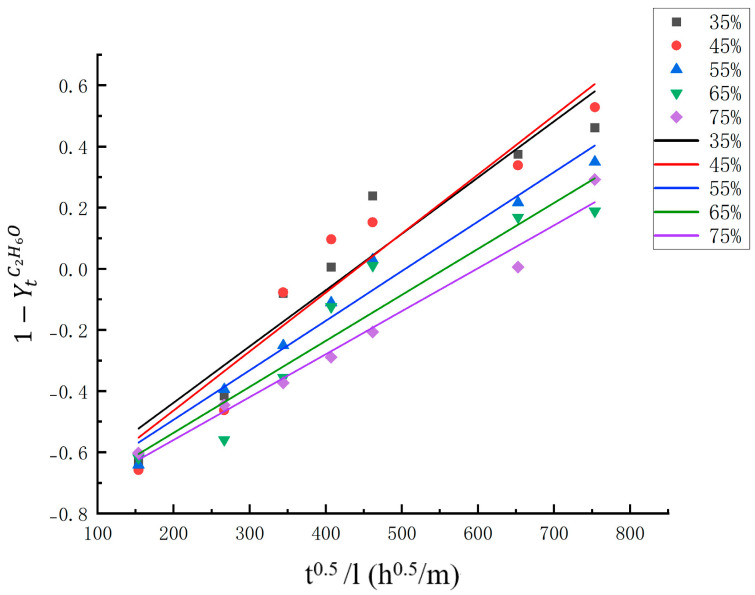
Mass transfer driving force 1-$Y_{t}^{C_{2}H_{6}O}\;$ regression curve with t^0.5^/L.

Fitted the experimental data according to Equstion (7) to obtain Figure 4 and Table 2. The diffusion coefficient represents the physical quantity of diffusion ability driven by concentration gradient, defined as the diffusion flux per unit concentration gradient, and is a subjective reflection of solute diffusion rate [14]. As indicated in the Table 2, the $D_{e}\;$ value is greater in solutions with lower ethanol concentrations. In the pickling process, changes are influenced not only by the chemical potential gradient but also by the structural changes in the tissue caused by the concentration of the pickling agent [34]. The highest $D_{e}$ value was observed in samples with an ethanol concentration of 45%, which was possibly because of the effect of ethanol on the microstructure. Higher ethanol concentrations lead to greater protein denaturation, thereby resulting in muscle contraction and surface hardening, which may increase resistance to molecular transfer and reduce the diffusion coefficient.

The diffusion of marinade is an important factor affecting the pickling effect, and the diffusion rate of marinade in the sample determines the pickling efficiency [10]. The effective diffusion coefficient values obtained in this study ranged from 1.53938 × 10^−6^$\;m^{2}/s$ to 2.83528 × 10^−6^$\;m^{2}/s$, which are significantly higher than those reported for saltwater-preserved meat products. According to the literature, the salt diffusion coefficients for salmon, sardine slices, and cod loins range from 10^−10^–10^−9^ (Wang et al. [16]; Andres et al. [9]; Barat et al. [6]; Gallart-Jornet et al. [15]; Nguyen et al. [35]). The complexity of driving forces (differences in water activity, pressure, and changes in protein structure) and the properties of different components, as well as the influence of alcohol on the microstructure of fibers, may be the reason for the observed higher $D_{e}$ values. Additionally, a high R^2^ value indicates a well linear correlation between the data and the formula fitting, which suggests that the analytical solution of Fick’s second law is suitable for mass transfer modeling in the processes of edible alcohol pickling.

### 3.3. Kinetic Parameters of Dealuminated Jellyfish During the Edible Alcohol Pickling Process

**Figure 5:**
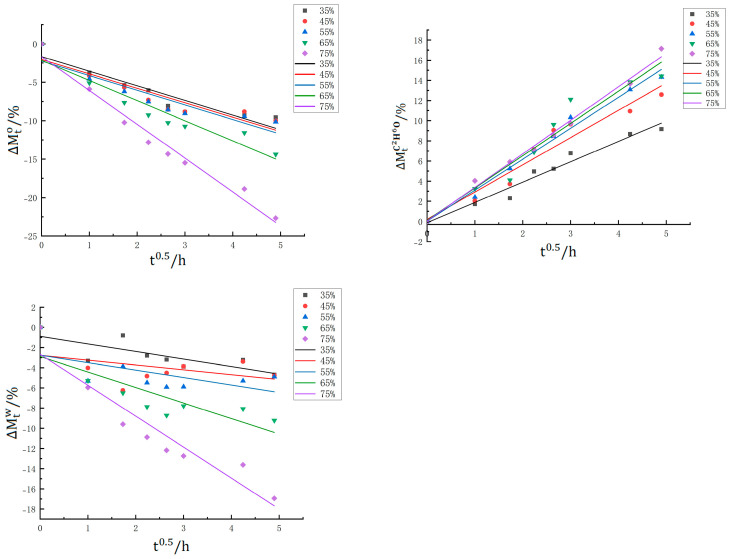
Regression curve between the total mass, ethanol content, and water content in jellyfish without alum and the square root of the pickling time.

The experimental data was calculated according to Formulas (1)–(3) and fitted with Formula (5), getted Figure 5 and Table 3. The Table 3 reveals that the weight changes and ethanol content changes in each concentration group are strongly correlated with the pickling time, this indicates that the model can be used to estimate the weight and alcohol content of dealuminated jellyfish at different pickling times. However, the R^2^ value for the water content changes is low. Individual differences among jellyfish, initial salt content, and water content variations in different parts of dealuminated jellyfish may contribute to the poor fit. In Equation (5), *k*_1_ describes the initial stage of the process, which is influenced primarily by the ethanol concentration, water activity gradient, and pressure gradient ([6,9]). The coefficient *k*_2_ is influenced mainly by the concentration of the pickling solution and is related to the process yield [6]. The *k*_2_ value for weight changes remained relatively consistent at ethanol concentrations from 35% to 55% but decreased significantly at ethanol concentrations of 65% and 75%, which indicates that the total weight of the jellyfish decreased more significantly with increasing pickling time at higher ethanol concentrations. Conversely, the *k*_2_ value for ethanol content changes increased with increasing ethanol concentration, as ethanol mass transfer is driven by the concentration gradients within the tissue and the pickling solution, thereby resulting in greater changes at higher ethanol concentrations.

### 3.4. Texture Change

Texture analysis primarily reveals the mechanical properties of food textures, with results that are highly sensitive and objective. These results can be accurately quantified to provide a comprehensive and objective evaluation of the food quality, thereby avoiding subjective biases in human factors [36]. As indicated in Table 4, the texture parameters of the jellyfish after dealumination decreased, where as those after soaking in ethanol significantly changed. Because ethanol serves as a polar organic solvent that interacts with charged proteins and affects their ordered structure, it is more conducive to the formation of a gel network structure [5]. After being soaked in 35% ethanol, the hardness and chewiness of the jellyfish still differed from those of edible jellyfish. After being soaked in 45% ethanol, the texture characteristics of the jellyfish were closer to those of edible jellyfish. In addition, with the increase in ethanol concentration (55~75%), there was no significant difference in the texture characteristics of the dealuminated jellyfish among different concentrations, thus indicating that higher concentrations of edible alcohol treatment did not significantly change the quality of the dealuminated jellyfish. One aspect to consider in the development of low-aluminum jellyfish is the definition of the final product characteristics, mainly the texture characteristics of the product [35]. Research has shown that the ethanol concentration that is suitable for dealuminated jellyfish is 45%.

### 3.5. Variation in the Aluminum Content

**Figure 6:**
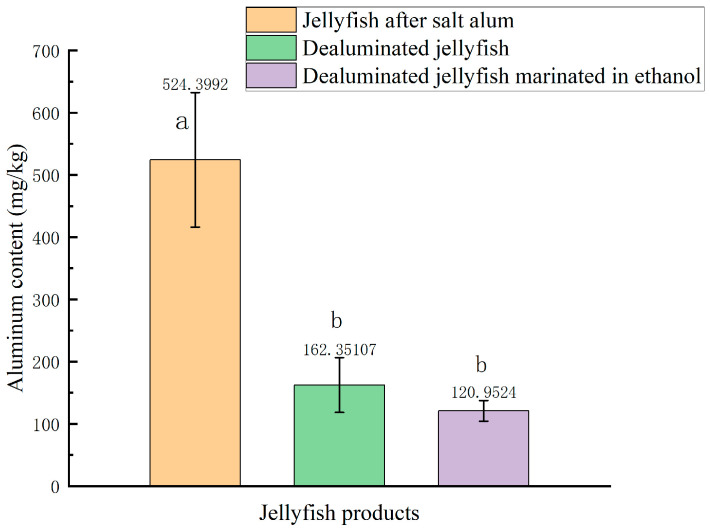
Aluminum contents of various jellyfish products. Note: a,b, indicate significant differences (*p* < 0.05).

The safety risks posed by excessive aluminum residues in jellyfish products have become a significant constraint on the development of the industry. Currently, industrial methods to reduce aluminum residues in salted jellyfish include soaking in clear water and using edible acid. However, these two methods often face issues such as time-consuming processes, poor results from clear water, and the softening of jellyfish and a decline in taste when treated with acidic solutions. Moreover, dehydrated jellyfish is more susceptible to bacterial and fungal contamination, leading to spoilage [37]. Lin [38] analyzed the microbial communities in five types of ready-to-eat jellyfish sold in the market and did not detect pathogenic bacteria such as Escherichia coli or Bacillus cereus in the three-month-old ready-to-eat jellyfish. However, the total bacterial count reached 4800 CFU/g, which seriously exceeded the standard for jellyfish. Alcohol has high permeability, its molecules can penetrate cell surfaces and enter protein chains, thereby causing proteins to denature and coagulate. This process not only causes protein dehydration and contraction but also yields antibacterial properties. Ethanol is safe and nontoxic, which makes it easy for people to accept, and can be added according to needs in food production [5]. Figure 6 shows the aluminum contents of various jellyfish products. GB2760-2024 (National Food Safety Standard for Food Additives) [39] specifies the aluminum content limit, which is ≤500 mg/kg for ready-to-eat jellyfish, ≤200 mg/kg for vermicelli, and ≤100 mg/kg for other types of food. The aluminum content in dealuminated jellyfish marinated with edible alcohol showed no significant difference compared to the aluminum content in dealuminated jellyfish, but decreased slightly, possibly due to further dissolution of residual aluminum ions, indicating that dealuminating jellyfish and then soaking it in ethanol is a safe method for dealuminating salted jellyfish, there by reducing the aluminum content while maintaining jellyfish quality.

## 4. Conclusions

This work demonstrates that using edible alcohol to treat dealuminated jellyfish maintains the texture of the product and improves its quality. This experiment provides data support for the improvement of the quality of jellyfish after aluminum removal, and provides a new perspective for the application of edible alcohol in the processing of aquatic products. In addition, product decomposition on the basis of the semi-infinite plate Fick equation and a short pickling time, as well as the pickling diffusion equation, was employed to describe the mass transfer behavior of dealuminated jellyfish in the pickling process. The results showed that the mass transfer model for various substances in the pickling process of dealuminated jellyfish exhibited a suitable linear correlation and could be used as a theoretical guide for ethanol pickling of dealuminated jellyfish. Therefore, according to the pickling equilibrium equation, if the mass ratio of jellyfish to pickling solution at the initial pickling stage, as well as the moisture and alcohol contents of the jellyfish and pickling solution at the initial stage, is known, the final alcohol content of the product can be estimated on the basis of the formula. The pickling equilibrium time can also be determined by comparing the theoretically calculated value with the actual measured value. This provides a key theoretical basis for the development of ethanol-soaked products and has the potential to promote innovation in green processing technology for jellyfish.

## Figures and Tables

**Table 1:** Theoretical equilibrium values and actual measured values after 48 h of pickling.

Ethanol Concentration	${Z_{e}}^{C_{2}H_{6}O}$	$Z^{C_{2}H_{6}O}$
35%	15.1%	16.0%
45%	21.2%	21.0%
55%	26.1%	25.8%
65%	31.1%	30.8%
75%	34.7%	34.6%

**Table 2:** Kinetic parameters (*De* and *K* values) obtained from Equation (7) and fitting correlation factors.

Ethanol Concentration	*D_e_* (m^2^/s) (10^−6^)	*K*	R^2^
35%	2.544690	−0.8058	0.9120
45%	2.835287	−0.8488	0.9182
55%	2.010619	−0.8181	0.9706
65%	1.767146	−0.8367	0.9059
75%	1.539380	−0.8408	0.9771

**Table 3:** Kinetic Dynamic parameter values and correlation coefficients of ethanol-soaked dealuminated jellyfish (${∆M}_{t}^{o},\;{∆M}_{t}^{C_{2}H_{6}O},and\;{∆M}_{t}^{w}$).

Variable	Ethanol Concentration	*k*_1_	*k*_2_	R^2^
	35%	−4.0755	−1.4840	0.8486
	45%	−4.7835	−1.3600	0.8234
$\Delta M_{t}^{o}$	55%	−5.1501	−1.3388	0.8658
	65%	−4.9893	−2.0771	0.9280
	75%	−4.1059	−3.9766	0.9779
	35%	−1.0898	1.9607	0.9432
	45%	−0.6096	2.6517	0.8996
$\Delta M_{t}^{C_{2}H_{6}O}$	55%	−0.8941	3.0422	0.9766
	65%	−0.7644	3.1676	0.9095
	75%	−0.8938	3.3122	0.9868
	35%	−1.3262	−1.3490	0.6905
	45%	−5.9763	0.2384	0.0943
$\Delta M_{t}^{w}$	55%	−5.8676	−0.1594	0.1011
	65%	−6.3404	0.8139	0.6878
	75%	−5.9346	−2.3981	0.9154

**Table 4:** TPA of dealuminated jellyfish under different ethanol concentrations ($\bar{x}$ ± SD).

Processing Method	Hardness (N)	Springiness	Chewiness	Gumminess	Cohesiveness
Ready-to-eat jellyfish	188.2 ± 20.6 ^bc^	1.10 ± 0.08 ^ab^	50.5 ± 12.8 ^bc^	45.6 ± 10.7 ^bcd^	0.28 ± 0.04 ^a^
Dealuminated jellyfish	140.8 ± 23.5 ^d^	1.03 ± 0.14 ^b^	29.8 ± 3.93 ^d^	28.9 ± 4.1 ^d^	0.2 ± 0.00 ^b^
35%	164.6 ± 18.5 ^cd^	1.00 ± 0.29 ^b^	40.3 ± 8.07 ^cd^	40.4 ± 8.5 ^cd^	0.27 ± 0.05 ^a^
45%	214.0 ± 39.7 ^ab^	1.00 ± 0.08 ^b^	44.1 ± 10.4 ^bc^	42.2 ± 10.1 ^bcd^	0.2 ± 0.00 ^b^
55%	219.7 ± 36.7 ^ab^	1.08 ± 0.11 ^ab^	60.5 ± 20.8 ^ab^	58.3 ± 24.4 ^abc^	0.25 ± 0.08 ^ab^
65%	232.2 ± 29.6 ^a^	1.18 ± 0.09 ^a^	78.3 ± 21.3 ^a^	67.3 ± 21.4 ^a^	0.27 ± 0.05 ^a^
75%	213.7 ± 18.6 ^ab^	1.16 ± 0.06 ^a^	71.6 ± 17.0 ^a^	60.9 ± 13.4 ^ab^	0.27 ± 0.05 ^a^

Note: The significance analysis results are shown in the same column, and a,b,c,d indicate significant differences (*p* < 0.05).

## Data Availability

The original contributions presented in this study are included in the article, and further inquiries can be directed to the corresponding author.
